# *BRAT1* gene compound heterozygous mutations causing lethal neonatal rigidity and multifocal seizure syndrome: a case report

**DOI:** 10.3389/fped.2026.1694328

**Published:** 2026-02-09

**Authors:** Dong-Yuan Qin, Qin-Qin Tang, Dan Feng, Yan-Jun Song, Zi-Huan Cheng, Rui-Cong Ma, Ke Sun, Fan Wang

**Affiliations:** Department of Neonatology, The Second Hospital & Clinical Medical School, Lanzhou University, Lanzhou, China

**Keywords:** aberrant splicing, *BRAT1* gene, compound heterozygous mutations, lethal neonatal rigidity and multifocal seizure syndrome, neonatal epilepsy, synonymous mutation, whole-exome sequencing

## Abstract

**Background:**

Biallelic BRCA1-associated ataxia telangiectasia mutated activator 1 (*BRAT1*) gene mutations can result in lethal neonatal rigidity and multifocal seizure syndrome (RMFSL), characterized by refractory epilepsy, hypertonia, autonomic dysfunction, and early death. This study reports an infant with RMFSL bearing novel compound heterozygous *BRAT1* gene mutations, including a rare pathogenic synonymous variant.

**Case presentation:**

A male infant born at 37 weeks of gestation presented with seizures shortly after birth. Clinical features included refractory epilepsy, bilateral clubfoot deformity, and respiratory failure. Whole-exome sequencing identified compound heterozygous *BRAT1* gene mutations (c.1395G>C, p.Thr465Thr and c.1297delC, p.Leu433Trpfs*). The c.1395G>C variant is a synonymous mutation with a predicted high-risk impact on mRNA splicing, whereas c.1297delC is a previously unreported novel frameshift mutation. These variants were inherited from phenotypically normal, healthy parents.

Despite the provided care, the infant died at one month of age.

**Conclusion:**

This case highlights that synonymous *BRAT1* variants affecting mRNA splicing can be pathogenic, leading to severe RMFSL. The findings expand the genotypic spectrum and underscore the need for comprehensive bioinformatics analysis of non-coding consequences in genetic testing.

## 1 Introduction

The BRCA1-associated ataxia telangiectasia mutated activator 1 (*BRAT1*) gene, located on chromosome 7 (7p22.3), encodes a protein that plays a critical role in DNA damage response through its interactions with BRCA1 and ataxia telangiectasia mutated (ATM) (1). Additional roles include p53-mediated apoptosis, cell growth signaling, and mitochondrial homeostasis (2). Biallelic *BRAT1* mutations result in either of two primary phenotypes: lethal neonatal rigidity and multifocal seizure syndrome (RMFSL; MIM#614498) or neurodevelopmental disorder with cerebellar atrophy and seizures (NEDCAS; MIM#618056) (1). RMFSL, which currently has no effective treatments, is characterized by refractory neonatal epilepsy, dystonia, autonomic instability, and infantile death (3). With recent advances in genetic testing, the phenotypic spectrum of *BRAT1*-related disorders has expanded. RMFSL usually arises in cases of biallelic nonsense, frameshift, or in-frame deletion/insertion variants (100% of cases), while NEDCAS typically has at least one missense variant (82% of cases). Splice variants exhibit variable phenotypes, with 41% presenting as RMFSL and 59% as NEDCAS. However, patients with RMFSL are rarely reported to have synonymous mutations, which are often presumed to be benign without comprehensive investigation of their potential impact on RNA splicing (4).

This study describes a Chinese neonate with epileptic encephalopathy in whom whole-exome sequencing revealed compound heterozygous mutations in the *BRAT1* gene (specifically the synonymous variant c.1395G>C, p.Thr465Thr and the novel frameshift variant c.1297delC, p.Leu433Trpfs*).

## 2 Case presentation

This study complies with the CARE checklist guidelines for case reports (Supplementary Materials) and was approved by the Ethics Committee of the Second Hospital of Lanzhou University. Informed consent was obtained from the parents.

### 2.1 Clinical manifestations

A male infant was admitted on day of life one for “recurrent seizure-like episodes after birth.” He was the second child (G2P1), delivered vaginally at 37 weeks +1 day of gestation, with a birth weight of 2.68 kg. Apgar scores were 9 (1 min, deducted for skin color) and 8 (5 min, deducted for skin color and response). The parents were healthy, non-consanguineous, and had no significant family history.

Shortly after birth, the infant exhibited limb rigidity, facial cyanosis, and seizures, which were temporarily alleviated by phenobarbital IV (30 mg/kg). However, seizures recurred, accompanied by persistent hypertonia, bilateral clubfoot deformity, and a simian crease on the right hand.

**Figure 1:**
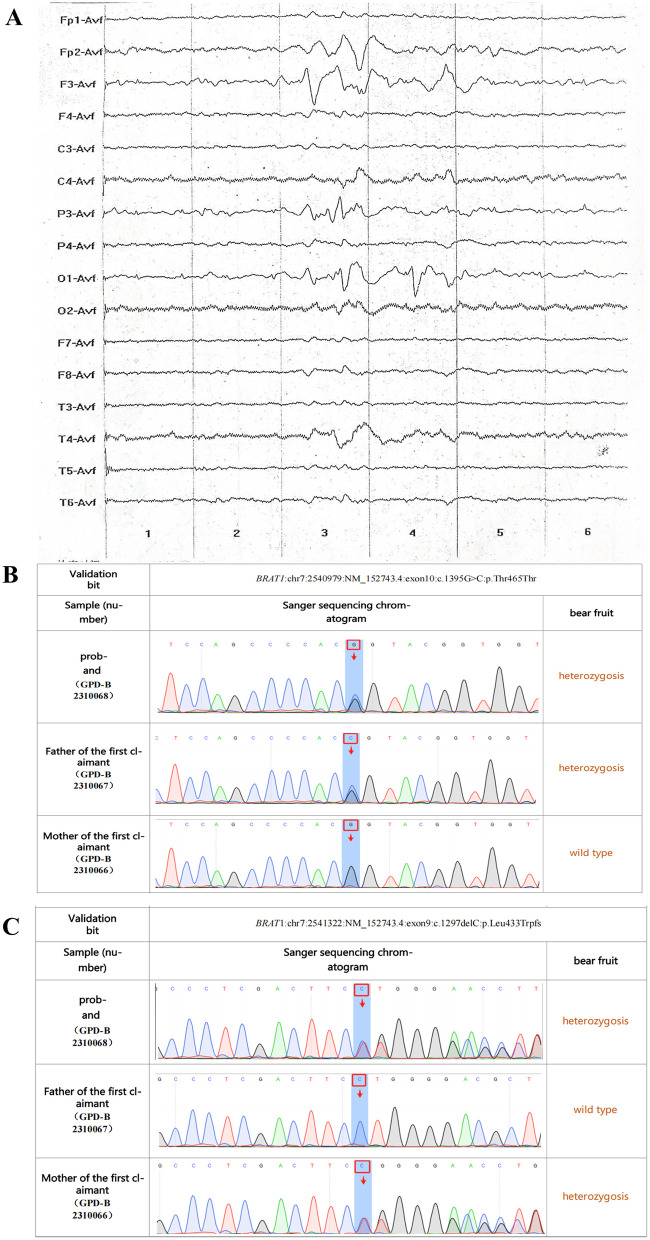
**(A)** Electroencephalography (2 days old) revealed 2–3 Hz sharp-slow waves in the left frontoparietal and occipital regions, with scattered slow waves in the right frontal and midtemporal regions. Sanger sequencing validation results schematic, with both **(B,C)** representing *BRAT1* variants. Top panel: Proband. Middle panel: Proband's father. Bottom panel: Proband's mother.

The infant required continuous non-invasive high-frequency oscillatory ventilation to treat respiratory failure and associated feeding difficulties. Complications included pulmonary infection (*Ureaplasma urealyticum* positive; chest X-ray demonstrated atelectasis and increased lung markings) and hypoalbuminemia (lowest albumin 22.1 g/L). EEG revealed 2–3 Hz sharp-slow waves in the left frontoparietal and occipital regions, with scattered slow waves in the right frontal and midtemporal regions (Figure 1A). Cranial ultrasound was remarkable for enhanced periventricular white matter signal. Given the refractory epilepsy, genetic testing was performed.

### 2.2 Genetic analysis

Whole-exome sequencing was performed using high-throughput next-generation sequencing with custom target capture probes. The average sequencing depth was 116.77×, with ≥20× coverage in 98.47% of regions. Sanger sequencing validated the candidate variants. Computational algorithms (PhyloP/GERP++, SIFT/PolyPhen-2/CADD/REVEL, SpliceAI/MaxEntScan, gnomAD/ExAC) predicted variant conservation, pathogenicity, and harmfulness. Variant classification followed the American College of Medical Genetics and Genomics (ACMG) guidelines.

The infant carried two pathogenic *BRAT1* variants:

1. c.1395G>C (p.Thr465Thr) (Figure 1B and Table 1): Inherited from the father, this variant, while synonymous for amino acid coding, was predicted to affect mRNA splicing (SpliceAI score >0.8). In the general population, it is rare (frequency 0.000193) and has been reported in patients with epileptic encephalopathy. It was also classified as pathogenic (PS3 + PM2 + PP3). PS3 was applied based on strong in silico evidence from SpliceAI and MaxEntScan, which predicted a disruptive impact on splicing. Such a splicing defect is considered functionally equivalent to a null variant for this gene (5). PM2 was applied due to its very low frequency in control populations. PP3 was assigned due to supportive computational evidence from multiple bioinformatics tools predicting a significant impact on the splice site.
2. c.1297delC (p.Leu433Trpfs*) (Figure 1C and Table 1): Inherited from the mother, this novel frameshift variant leads to protein truncation and is absent from population databases (e.g., GnomAD). It was classified as pathogenic (PVS1 + PM2 + PP3) according to ACMG guidelines. PVS1 was applied as it is a null variant (frameshift) in a gene where loss of function is a known mechanism of disease (6). PM2 was applied due to its absence from population databases. PP3 was assigned based on concordant computational evidence supporting a deleterious effect from multiple prediction tools.

**Table 1:** Pathogenic variants in BRAT1 (c.1395G>C and c.1297delC).

Gene	Chromosomal location (hg38)	Reference transcript and exon	Nucleotide change	Amino acid change	Functional consequence	Zygosity	Inheritance	Population frequency (gnomAD)	Pathogenicity interpretation
*BRAT1*	chr7:2540979	NM_152743.4 exon 10	c.1395G>C	p.Thr465Thr	Synonymous variant	Heterozygous (82/162)	Paternal	0.000193	Pathogenic
*BRAT1*	chr7:2541322	NM_152743.4 exon 9	c.1297delC	p.Leu433Trpfs	Frameshift variant	Heterozygous (83/165)	Maternal	Not reported	Pathogenic

Both variants were therefore classified as pathogenic, confirming the molecular diagnosis of an autosomal recessive disorder.

### 2.3 Treatment and follow-up

The infant received triple antiepileptic pharmacotherapy: phenobarbital IV (20 mg/kg loading dose, then 5 mg/kg/day), levetiracetam IV (40 mg/kg/day), and midazolam infusion (0.1–0.3 μg/kg/min). However, clinical seizures persisted, and EEG showed uncontrolled discharges. Additional therapy included azithromycin (10 mg/kg/day for 5 days) for *U. urealyticum* infection, albumin infusion (1 g/kg/day for 3 days) for hypoalbuminemia, and furosemide (1 mg/kg q12 h) for edema. Despite aggressive medical management, the infant remained ventilator-dependent, with frequent seizures and feeding intolerance. Genetic testing confirmed *BRAT1*-related RMFSL, as described previously. The parents chose to pursue supportive palliative care, and the infant died shortly after discharge.

## 3 Discussion

In 2012, Puffenberger et al. first confirmed the association of biallelic *BRAT1* mutations with RMFSL (7). *BRAT1* mutations can cause two phenotypes: severe RMFSL (100% mortality by age 3) and milder NEDCAS (longer survival, 76% ambulatory) (4, 8). RMFSL is associated with biallelic loss-of-function (LOF) variants, while NEDCAS often involves missense variants (4).

Our patient exhibited classic RMFSL features, including refractory epilepsy, hypertonia, clubfoot, respiratory failure, and early death. The c.1297delC variant, while not previously reported, represents a typical LOF mutation. The c.1395G>C variant represents a more nuanced but equally critical mechanism of pathogenicity. Although synonymous at the protein level (p.Thr465Thr), this variant is a powerful example of how a single nucleotide change that does not alter the amino acid code can still cause severe disease by disrupting the intricate process of mRNA splicing. The SpliceAI score of >0.8 indicates a high probability of altered splicing, a threshold commonly associated with clinically significant splice-altering variants (9). This prediction was further supported by complementary in silico tools employed in our analysis (e.g., MaxEntScan), which likely predicted a significant reduction in the strength of the native splice site or the creation of a cryptic splice site. The concordance of multiple bioinformatics tools strengthens the evidence that c.1395G>C is not a silent polymorphism but a splice-disrupting variant. Similar cases—such as the synonymous variant c.1014A>C (p.Pro338=), which also likely affects splicing—have been reported (10). Therefore, both identified variants are expected to result in a complete loss of functional BRAT1 protein, consistent with the severe RMFSL phenotype observed. This case underscores the point that the traditional assumption of synonymous variants being benign is outdated; they can be pathogenic through mechanisms that evade detection by standard protein-centric analyses.

The formal ACMG classifications (PS3 + PM2 + PP3 and PVS1 + PM2 + PP3) underscore the pathogenic role of both alleles (5).

The profound therapeutic resistance observed in BRAT1-related RMFSL, exemplified by the failure of combination therapy with phenobarbital, levetiracetam, and midazolam in our patient, can be understood by considering the fundamental cellular functions of the BRAT1 protein. Unlike the mechanisms targeted by most conventional antiseizure medications (ASMs)—which primarily modulate synaptic transmission, ion channels, or neurotransmitter levels—the pathophysiology of BRAT1-related disorders originates from profound dysfunction in core cellular homeostasis. BRAT1 is integral to DNA damage response and repair pathways through its interactions with BRCA1 and ATM (1, 2). Its deficiency leads to genomic instability and impaired p53-mediated apoptosis, likely contributing to aberrant neuronal survival and circuit formation. Perhaps more critically for excitability, BRAT1 is essential for maintaining mitochondrial function (2). Mitochondrial dysfunction disrupts adenosine triphosphate production, calcium buffering, and reactive oxygen species regulation, creating a cellular environment prone to hyperexcitability that lies entirely upstream of traditional synaptic targets. Therefore, the intrinsic refractoriness of BRAT1-related epilepsy is not due to pharmacoresistance in the classical sense, but rather because conventional ASMs do not address these foundational cytopathological deficits. Phenotypic variability in BRAT1-related disorders is likely determined by mutation type and, more importantly, the degree of residual protein function. Biallelic LOF variants cause RMFSL, whereas missense or splice variants may retain partial function, resulting in NEDCAS (11, 12). In our patient, the bioinformatically predicted splicing defect caused by c.1395G>C is presumed to have abolished BRAT1 function, in combination with the truncating c.1297delC mutation, resulting in severe RMFSL. This case exemplifies the principle that the functional consequence of a variant (complete loss-of-function), rather than its precise molecular type (synonymous vs. truncating), is the primary determinant of phenotypic severity.

Diagnostic challenges in BRAT1-related disorders include phenotypic heterogeneity, undetected deep intronic variants, and non-neurological features (e.g., clubfoot), complicating diagnosis (4, 13, 14). This case highlights another critical challenge: the under-recognition of pathogenic synonymous variants. As this case demonstrates, genetic testing is crucial, and the interpretation must extend beyond the exonic code to include rigorous in silico analysis of potential splicing impacts for all variants, particularly synonymous ones, which are often erroneously filtered out (15). It is important to note that current management remains entirely supportive, as epilepsy in RMFSL is notoriously refractory to standard treatments. In this case, and consistent with other reports, aggressive combination therapy with phenobarbital, levetiracetam, and continuous midazolam infusion failed to achieve seizure control. Given the mechanistic insights mentioned previously, it is evident that current treatment remains purely supportive and ineffective at altering the course of the disease. This finding underscores the urgent need for future research to move beyond synaptic modulation and explore truly targeted therapeutic strategies. These include small molecules designed to modulate the integrated stress response or enhance mitochondrial biogenesis, or the use of gene-based therapies to restore BRAT1 function, thereby directly addressing the root cause of this disorder (2).

## 4 Conclusion

RMFSL currently has no effective treatment, necessitating further research for early diagnosis and therapy optimization. This case expands the *BRAT1* genotype–phenotype spectrum in Chinese populations. Infants with refractory epilepsy with hypertonia should be tested for *BRAT1* mutation, facilitating accurate diagnosis and genetic counseling to help manage parental expectations. Furthermore, this report highlights the critical importance of scrutinizing synonymous variants with advanced bioinformatics tools to assess their potential impact on splicing. Future studies should elucidate the mechanisms of *BRAT1* and develop targeted therapies.

## Data Availability

The raw data supporting the conclusions of this article will be made available by the authors, without undue reservation.
